# Optimizing Road Safety: Advancements in Lightweight YOLOv8 Models and GhostC2f Design for Real-Time Distracted Driving Detection

**DOI:** 10.3390/s23218844

**Published:** 2023-10-31

**Authors:** Yingjie Du, Xiaofeng Liu, Yuwei Yi, Kun Wei

**Affiliations:** School of Automotive and Transportation, Tianjin University of Technology and Education, Tianjin 300222, China; xfliu@tute.edu.cn (X.L.); 0721211006@tute.edu.cn (Y.Y.); 2019120012@tute.edu.cn (K.W.)

**Keywords:** attention mechanism, distracted driving, feature fusion, GhostConv, YOLOv8n

## Abstract

The rapid detection of distracted driving behaviors is crucial for enhancing road safety and preventing traffic accidents. Compared with the traditional methods of distracted-driving-behavior detection, the YOLOv8 model has been proven to possess powerful capabilities, enabling it to perceive global information more swiftly. Currently, the successful application of GhostConv in edge computing and embedded systems further validates the advantages of lightweight design in real-time detection using large models. Effectively integrating lightweight strategies into YOLOv8 models and reducing their impact on model performance has become a focal point in the field of real-time distracted driving detection based on deep learning. Inspired by GhostConv, this paper presents an innovative GhostC2f design, aiming to integrate the idea of linear transformation to generate more feature maps without additional computation into YOLOv8 for real-time distracted-driving-detection tasks. The goal is to reduce model parameters and computational load. Additionally, enhancements have been made to the path aggregation network (PAN) to amplify multi-level feature fusion and contextual information propagation. Furthermore, simple attention mechanisms (SimAMs) are introduced to perform self-normalization on each feature map, emphasizing feature maps with valuable information and suppressing redundant information interference in complex backgrounds. Lastly, the nine distinct distracted driving types in the publicly available SFDDD dataset were expanded to 14 categories, and nighttime scenarios were introduced. The results indicate a 5.1% improvement in model accuracy, with model weight size and computational load reduced by 36.7% and 34.6%, respectively. During 30 real vehicle tests, the distracted-driving-detection accuracy reached 91.9% during daylight and 90.3% at night, affirming the exceptional performance of the proposed model in assisting distracted driving detection when driving and contributing to accident-risk reduction.

## 1. Introduction

Distracted driving behavior has led to a large number of accidents and casualties. According to the World Health Organization (WHO), up to 1.3 million deaths are caused by traffic accidents each year, and traffic accidents have become the leading cause of death among young people [1]. According to data from the U.S. National Highway Traffic Safety Administration (NHTSA), in 2020, 3142 people died in motor vehicle accidents caused by distracted driving [2]. These accidents not only cause serious damage to people and property but also impose a huge financial burden on individuals and families [3]. There are numerous factors that contribute to distracted driving; however, distraction due to simultaneous multi-tasking while driving has been identified as the main cause [4]. In addition, when a driver engages in distracted driving, they not only increase their own risk of accidents but also elevate the risk for other drivers, pedestrians, and cyclists. The probability of traffic accidents increases as the number of vehicles in traffic increases. Therefore, by enhancing the speed and accuracy of the distracted-driving-behavior-detection algorithm, the assistance system can warn the driver or take necessary actions earlier, thereby reducing the risk of accidents [5]. This is of significant importance for improving road safety. Furthermore, studying distracted driving behaviors is of significant importance for traffic management departments in formulating relevant regulations and enhancing drivers’ safety awareness [6].

Previous studies have primarily focused on two methods: internal feature detection [7,8] and computer vision detection [9,10]. The former requires attention to the driver’s physiological and psychological states [11], such as eye movement, brain waves, and heart rate. However, the collection and analysis of physiological signals may necessitate specific equipment and techniques, and due to significant individual differences, disparities in results among different drivers may arise. Therefore, computer vision detection, which offers a more intuitive and concrete approach, has been widely applied in research into distracted driving.

In the early days, traditional machine learning methods identified distracted and non-distracted driving behaviors by manually designing features and selecting appropriate classification models [12,13]. Ahangari et al. [14] achieved an accuracy of 76.5% on an independent test set collected from a driving simulator using a random forest (RF) classifier. Gao et al. [15] used time-window and fast Fourier transform methods to obtain the vehicle dynamics parameters required for the model and achieved 85.68% interference discrimination accuracy using the XGBoost model. However, these methods usually rely on manually designed features and may fail to adequately capture complex patterns of distraction. In addition, there are difficulties in data acquisition which may result in unbalanced data distribution, which constrains the generalization performance of the model. Therefore, to overcome these challenges, deep learning methods enabling automatic feature learning have been introduced to cope with complex distracted driving behaviors and enhance model robustness.

With the widespread application of deep learning methods in research into distracted driving [16], an increasing number of researchers have begun to establish their own datasets for studying distracted driving behaviors. Jin et al. [17] achieved an accuracy of up to 95.7% on their self-constructed dataset by optimizing the size and quantity of convolutional kernels in a convolutional neural network. While this method excels in the aspect of feature extraction, the global pooling operation it employs may lead to a loss of spatial information, consequently diminishing the ability to perceive the precise location of the target.

The two-stage object detection algorithm first generates candidate regions in the image, and then employs a classifier for classification, thereby more accurately recovering target positional information. Yan et al. [18] utilized a Gaussian mixture model to extract skin-like regions, enabling R-CNN to achieve an accuracy of 97.76% on the Southeast University Distracted Pose (SUE-DP) dataset, which includes four types of distracted driving behaviors. Relying on the Faster R-CNN deep learning model developed by Seshadri et al., Ren et al. [19] conducted experiments on the dataset, achieving an impressive accuracy of up to 94.2%. However, these methods exhibit a high level of complexity and involve numerous hyperparameters, leading to a relatively slow processing speed. Therefore, Ghiasi et al. [20] proposed a single-stage YOLO algorithm. Its uniqueness lies in its ability to simultaneously predict multiple bounding boxes along with their corresponding class probabilities. Compared to traditional two-stage algorithms, the YOLO algorithm demonstrates remarkable processing speed. By transforming the object detection task into a regression problem, it accomplishes the simultaneous classification and position prediction of targets in a single step. Therefore, the YOLO algorithm has been widely applied in the field of distracted-driving-behavior detection. Murthy et al. [21] employed YOLOv5s as the detection framework in the driver-assistance system, and its detection performance surpassed that of YOLOv3 and YOLOv4 models. Regrettably, they overlooked the vital use of lightweight methods in real-time detection and analysis of driving behavior. Jing Wang et al. [22] innovated a lightweight convolutional neural network (CNN) approach, effectively enhancing the overall inference speed by 1.5 times through optimizing the block layers and reducing network channels. Zhao et al. [23] adopted the Inception V3 architecture while incorporating convolutional block attention and squeeze-and-excitation modules to extract crucial feature information. This achieved a balance between precision and speed. After acceleration, the average detection time on the Raspberry Pi 4B was reduced to 197 milliseconds. While these methods achieve balance between accuracy and speed, they fall short in enhancing detection for diverse target scales. Li et al. [24] merged attention mechanisms with bidirectional feature pyramid networks, boosting the model’s capacity to amalgamate diverse-scale object data. This led to a remarkable 95.6% accuracy on the SFDDD dataset, albeit accompanied by an extra computational load. Yolov5 [25] adopts a backbone network structure based on an FPN (feature pyramid network) and uses an anchor-free detection method, reducing the model’s computational load and the number of parameters, but with lower detection performance. Yolov7 is a new object detection model [26]. Compared to Yolov5, it introduces E-ELAN with an expand, shuffle, and merge cardinality structure, enhancing the network’s learning capability without disrupting the original gradient path [27]. YOLOv8 abandons the previous anchor-based method and adopts an anchor-free concept [28]. It also introduces the C2f module, enhancing the model’s feature-extraction capability, resulting in further improvements in accuracy.

In summary, prior research has achieved significant accomplishments in the detection of distracted driving behavior. However, despite some attempts at lightweight optimization, the computational requirements and parameter complexity of current models are still relatively large and cannot simultaneously balance the detection performance for targets at different scales. Furthermore, the existing SFDDD [29] and AUC [30] datasets lack representation of distracted behaviors such as yawning, wiping glass, smoking, voicing right, and voicing left, and similar activities during driving. Equally noteworthy is the absence of consideration for diverse environmental factors between daytime and nighttime, alongside real-world vehicular validation scenarios.

Therefore, this paper proposes a lightweight detection model named YOLO-LBS, which not only pursues lightweight design but also balances detection accuracy. Firstly, by integrating the GhostConv and YOLOv8n architectures, a lightweight network is devised. Additionally, a novel GhostC2f structure is introduced to substantially alleviate computational load and reduce parameter count. GhostC2f can generate more feature maps through linear transformations without requiring additional computation. Secondly, the path aggregation network (PAN) is optimized to improve the model’s feature-fusion capability. Subsequently, the simple attention mechanism (SimAM) is incorporated into the detection layers of the original network, strengthening the detection head’s capacity for extracting and localizing relevant information within the network. Lastly, various data-augmentation techniques are implemented, encompassing random translation, modifications in brightness and saturation, and the introduction of noise. The objective is to expand the initial collection of nine instances of distracted driving behaviors from the SFDDD dataset to a comprehensive total of 14 behaviors. This augmentation also incorporates scenarios occurring during nighttime. The credibility of these augmentations is verified via real vehicle experiments.

The remaining sections are structured as follows. The framework of the YOLO-LBS model is proposed in Section 2. Section 3 and Section 4 present the experiment design and case study. Section 5 offers some thoughts on the findings.

## 2. Materials and Methods

### 2.1. YOLOV8n Network Structure

**Figure 1:**
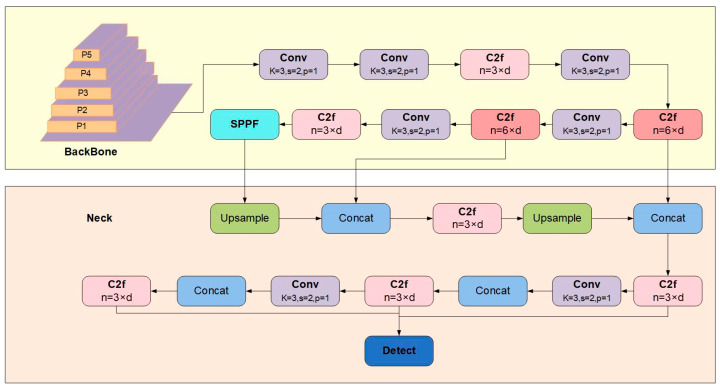
Structure diagram of YOLOv8n.

YOLOv8 is a single-stage object detection algorithm based on regression techniques, aimed at further enhancing detection performance. This model encompasses four distinct network structures: YOLOv8n, YOLOv8m, YOLOv8l, and YOLOv8x. These architectures have been bolstered in terms of both depth and breadth to improve detection accuracy, albeit at the cost of increased computational complexity. Given the high-speed responsiveness required for distracted-driving detection, we selected the YOLOv8n model, renowned for its swiftness, as the baseline and subsequently refined its optimization. The YOLOv8s model is comprised of four crucial components: the input layer, the backbone network layer, the neck structure, and the output layer, as illustrated in Figure 1.

The input layer’s primary role involves receiving input images and priming them for subsequent processing. This preparation includes tasks like mosaic data augmentation, adaptive anchor box calculation, and adaptive grayscale padding.

The backbone section serves the purpose of feature extraction and encompasses modules such as Conv, C2f, and SPPF. Within the Conv module, operations encompass convolution, batch normalization (BN), and SiLU activation functions. YOLOv8n introduces an innovative C2f structure designed to acquire residual features, thereby upholding gradient-flow information while ensuring a lightweight design. Meanwhile, the SPPF module, known as spatial pyramid pooling, converts the feature map into a feature vector of fixed dimensions.

The neck structure establishes a connection between the backbone network and the output layer, facilitating the integration and fusion of features across diverse scales and abstraction levels. Employing FPN and PAN structures, the neck section captures contextual information to enhance the representation proficiency of objects differing in size and position.

At the output layer, the generation of object detection predictions takes place. This step involves the application of non-maximum suppression (NMS) techniques to filter out redundant outcomes, thereby preserving dependable and precise prediction results.

### 2.2. Improved YOLO-LBS Network Structure

#### 2.2.1. Lightweighting Improvements

**Figure 2:**
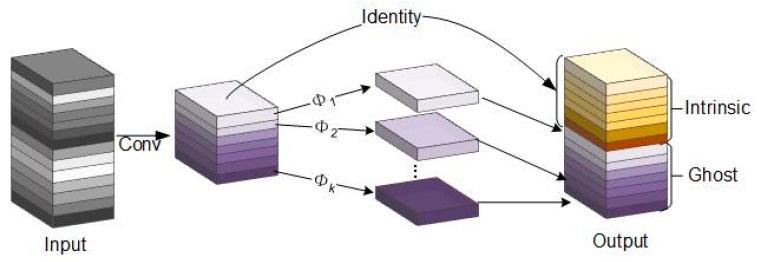
GhostConv structure.

Detecting distracted driving behavior accurately and promptly is critical for preventing accidents. However, the YOLOv8n models’ feature extraction relies mainly on 3 × 3 convolutional operations, leading to increased model parameters and computational costs, unsuitable for rapid detection. Thus, this study adopts the streamlined GhostConv network [31] for optimization. GhostConv initiates preliminary feature extraction on input feature maps using a small number of convolutional kernels, followed by a concise linear transformation. Concatenation operations then generate the final feature map, illustrated in Figure 2.

This study applies GhostConv to optimize the YOLOv8n model’s backbone and neck networks’ convolutional layers by retaining the original convolutional layers’ receptive field and feature extraction, model parameters, and complexity decrease, catering to resource-limited environments, enhancing real-time performance, and reducing storage.

**Figure 3:**
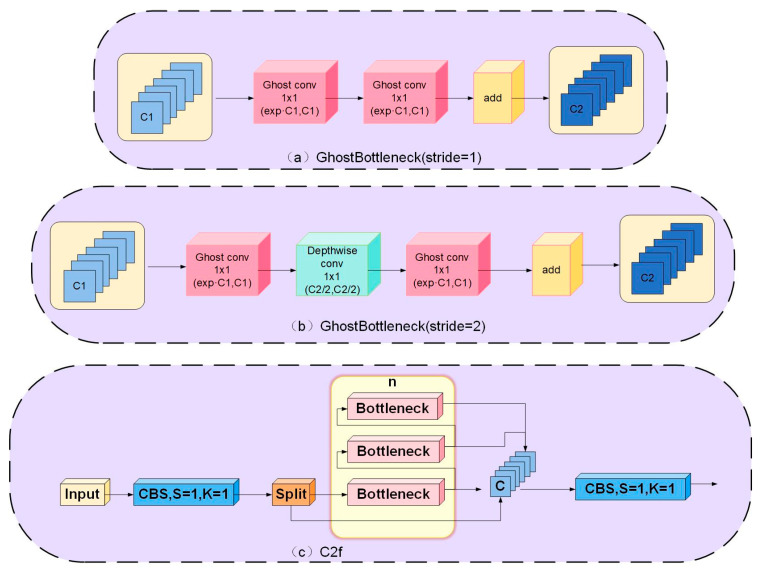
Two lightweight constructions and C2f construction. (**a**) GhostBottleneck (stride = 1) construction; (**b**) GhostBottleneck (stride = 2) construction; (**c**) C2f construction.

The lightweight GhostConv-based convolutional structure, GhostBottleneck, offers two options: stride 1 and stride 2. The stride 1 structure includes two 1 × 1 GhostConv layers and a residual link. The first layer widens the feature map’s channels, and the second restores channels, matching input channels, as in Figure 3a. The stride 2 structure adds depthwise separable convolution atop stride 1 alongside 1 × 1 convolution for subsampling and restoration. This maintains coherence between residual branches and input features, reducing gradients, as shown in Figure 3b.

**Figure 4:**
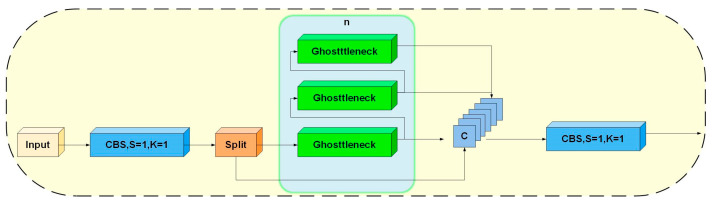
GhostC2f structure.

Although YOLOv8n primarily deploys C2f for feature extraction, C2f contains multiple bottleneck layers, as seen in Figure 3c. Each bottleneck includes various convolutional kernels—1 × 1, 3 × 3, and 1 × 1—resulting in multiple parameters and high complexity. Hence, this study revamps the Bottleneck structure, replacing it with GhostBottleneck layers, substantially reducing parameters, simplifying models, and enhancing detection. GhostBottleneck employs channel grouping for feature extraction, aiding the model’s diverse feature learning, as illustrated in Figure 4.

#### 2.2.2. Bidirectional Feature Pyramid Network (BiFPN)

**Figure 5:**
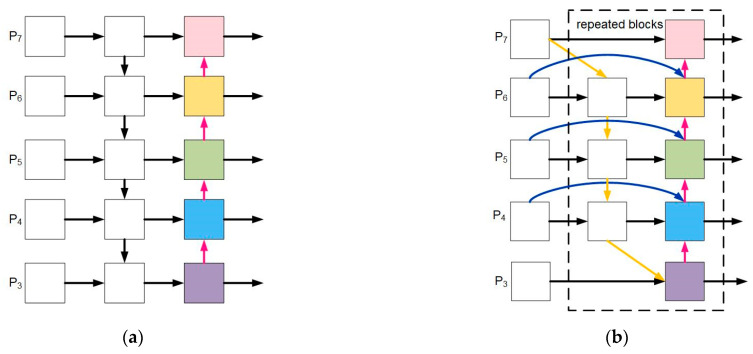
Structure diagram of PAN and BiFPN. (**a**) Structure of PAN; (**b**) structure of BiFPN.

In the actual process of driving, differences in drivers’ body shapes and variations in the installation angles of the collection equipment can result in varying dimensions of the detected objects. To address this issue, the neck network layer of YOLOv8n adopts the FPN + PAN architecture for processing feature information. FPN [23] handles semantic features from top to bottom, while PAN facilitates the transmission of positional information from bottom to top. Despite the FPN + PAN architecture’s incorporation of both semantic and positional information, the model’s parameters are relatively extensive, leading to a lower efficiency in fusing effective information. Therefore, this study enhances the integration and utilization of feature pyramids of varying scales through the improved BiFPN [32], aiming to enhance the model’s perceptual capability and information-extraction efficiency for objects of different sizes. Its structure is shown in Figure 5.

**Figure 6:**
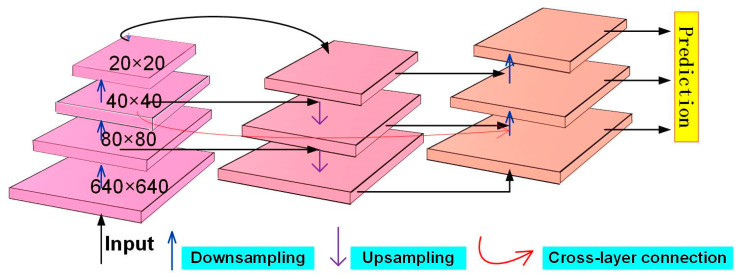
The BiFPN structure.

Specifically speaking, firstly, building upon the PAN structure, it was observed that certain nodes possessed unidirectional inputs without featuring any element of feature fusion. These nodes contributed relatively less to the feature-fusion process while introducing additional computational load and parameters. To enhance the efficiency of object detection, a strategy was employed, namely, the removal of nodes with lower contributions. Secondly, within the same channel, the concept of skip channels was introduced between the input and output nodes. This allowed each pair of bidirectional pathways to be treated as a feature layer, enabling more comprehensive information fusion. This approach effectively captured more fusion information while maintaining a controllable computational load. The proposed improved structure is illustrated in Figure 6.

Considering that input features of different resolutions might influence the output features to varying degrees, to fuse these features effectively, BiFPN introduced a weighted feature fusion strategy. In this method, a relevance weight was introduced for each feature. These weights were subsequently normalized for utilization in feature fusion. This weighting mechanism ensured that weight values ranged from 0 to 1. The specific calculation process is referred to in Equation (1):

(1) $$O=\sum_{i}\frac{w_{i}}{\varepsilon +\sum_{j}w_{j}}\cdot I_{i}$$

where O denotes the output features, $I_{i}$ represents the input features, and w signifies the node weights. It is noteworthy that the learning rate ε is set to 0.0001, with the purpose of preventing the generation of unstable outcomes. 

#### 2.2.3. Similarity-Based Attention Mechanism

In real driving scenarios, the diverse backgrounds of drivers make the accurate identification of distracted driving behavior a considerable challenge. To more effectively extract crucial information under such circumstances, attention mechanisms have been extensively employed in the study of distracted driving behavior. Nevertheless, current attention mechanisms commonly encounter two issues. Firstly, they are constrained to refining features along the channel or spatial dimensions, thereby limiting their ability to simultaneously learn weights across different channels. Secondly, in pursuit of high performance, these mechanisms often require the adjustment of certain hyperparameters, leading to an increase in the model’s parameter count. 

**Figure 7:**
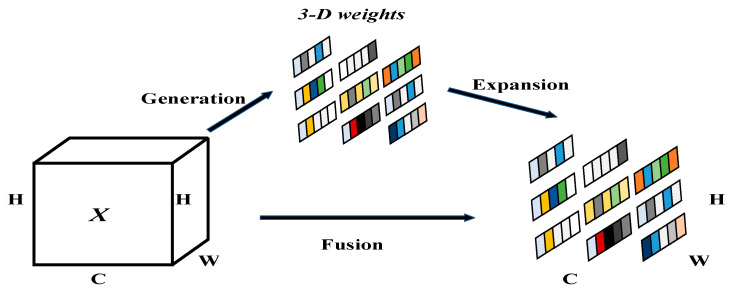
Similarity-based attention mechanism structure.

To address these concerns, this study introduces three SimAMs (similarity-based attention mechanisms) at the detection layer, as illustrated in Figure 7. Inspired by theories of neural science, a SimAM utilizes an energy function to compute weights for feature extraction. It can infer three-dimensional attention weights without introducing additional network parameters. These three-dimensional attention weights are presented in Figure 7.

By employing the energy-based attention mechanism, SimAM effectively computes the 3D attention weights. In comparison to alternative attention mechanisms, SimAM adeptly circumvents the challenge of escalating model parameters due to structural adjustments, thereby significantly amplifying the efficacy of distracted-driving-behavior detection. Representation of the energy function for each individual neuron is articulated as Equation (2):

(2) $$\begin{array}{c} e_{t}\left(w_{t}t,b_{t},y,x_{i}\right)=\frac{1}{M-1}\sum_{i=1}^{M-1}{\left[-1-\left(w_{i}x_{i}+b_{t}\right)\right]}^{2}\\ +{\left[1-\left(w_{t}t+b_{t}\right)\right]}^{2}+\lambda w_{i}^{2} \end{array}$$

where t, i, and x correspondingly signify the location of the target neuron, the index of the spatial dimension, and the remaining neurons within the input tensor X on a solitary channel. M signifies the total count of neurons in a specific channel, while y denotes the measure of neuronal significance. The precise formulations for the weight $w_{t}$ and bias $b_{t}$ of the energy function are provided by Equations (3) and (4):

(3) $$w_{t}=-\frac{2\left(t-\mu_{t}\right)}{{\left(t-\mu_{t}\right)}^{2}+2\delta_{t}^{2}+2\lambda }$$

(4) $$b_{t}=-\frac{1}{2}\left(t+\mu_{i}\right)w_{t}$$

where $u_{t}$ and $\delta_{t}^{2}$, respectively, describe the mean and variance of neurons within the channel excluding the target neuron, as shown in Equations (5) and (6):

(5) $$\mu_{t}=\frac{1}{M-1}\sum_{i=1}^{M-1}x_{i}$$

(6) $$\delta_{t}^{2}=\frac{1}{M-1}\sum_{i=1}^{M-1}{\left(x_{i}-\mu_{t}\right)}^{2}$$

Specifically, SimAM calculates energy and infers that when the energy of a particular neuron is lower, its dissimilarity with other neurons is greater, indicating its higher significance. Consequently, the SimAM attention mechanism accurately captures key information within image features without necessitating the introduction of extra parameters, thereby holding substantial practical application value.

## 3. Experiments and Results

### 3.1. Dataset Augmentation

**Figure 8:**
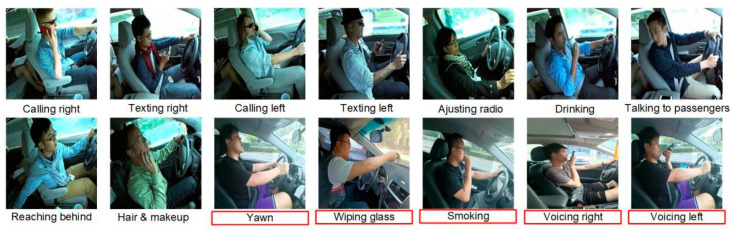
Fourteen distracted driving behaviors.

With the proliferation of smartphones and various intelligent in-car multimedia systems and entertainment functions, drivers are required to access and process a greater amount of information during the driving process. Currently, the publicly available State Farm Distracted Driver Detection (SFDDD) dataset comprises images of nine types of distracted behaviors (at a resolution of 640 × 480), including calling right, texting right, calling left, texting left, adjusting radio, drinking, talking to passengers, reaching behind, and hair and makeup. However, this dataset fails to account for the following five types of attention-diverting behaviors: yawning, wiping glass, smoking, voicing right, and voicing left, which are highlighted in red in Figure 8. Additionally, nighttime scenes are not encompassed. This oversight could potentially result in the model’s inability to effectively identify newly emerging or evolving distracted driving behaviors.

To address this gap, the present study leverages the publicly available SFDDD dataset to construct a novel dataset named Diverse Distracted Driving (DDD). This dataset encompasses fourteen types of daytime distracted driving behaviors, as illustrated in Figure 8. Data collection was conducted using the rear camera of a Huawei 20 smartphone, with the following parameters: image resolution of 3000 × 4000, focal length of 2.0, and a capture pitch angle ranging from 45° to 60°. These measures are anticipated to enhance the model’s performance in recognizing distracted driving behaviors, thereby enabling it to respond to newly emerging or evolving driving scenarios more accurately.

**Figure 9:**
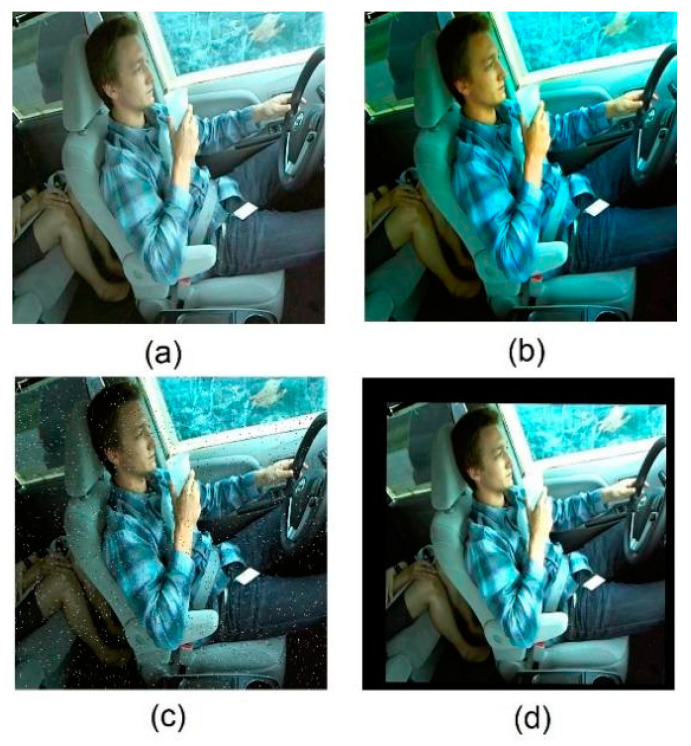
Data-enhancement diagram. (**a**) Adjusting brightness, (**b**) adjusting saturation, (**c**) adding noise, and (**d**) random panning.

To boost the model’s generalization ability, we enriched the dataset with diverse augmentation techniques—adjusting brightness, saturation, adding noise, and random translations. This expanded the dataset to 3300 samples (see Figure 9). Distracted driving behaviors were labeled using text files. These files had five columns: label category, label-box center’s x and y coordinates, width, and height.

**Figure 10:**
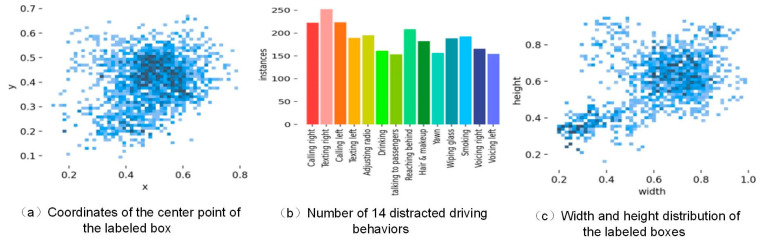
Label data volume and label distribution of distracted driving behaviors.

The dataset was divided into an 8:2 ratio for training and validation sets. In the training set, the data distribution across 14 distracted-driving-behavior categories and the details of the label boxes are shown in Figure 10.

### 3.2. Experimental Environment and Assessment Indicators

In the Linux operating system environment, experiments were conducted utilizing the Intel Xeon CPU E5-2680 v3, NVIDIA GeForce RTX 2080 Ti GPU with 11 GB VRAM, PyTorch framework version 1.7.0, and Python version 3.8. The experiment employed the following set of hyperparameters: an initial learning rate of 0.01, a total of 1100 training epochs, momentum set to 0.937, weight loss coefficient of 0.0005, and a batch size of 16.

To ensure a more precise assessment of object detection performance, four essential metrics were introduced: precision (P), recall (R), F1, and mean average precision (mAP) [33,34]. The specific computational formulas for these metrics are provided as follows (Equations (7)–(10)):

(7) $$P=\frac{TP}{TP+FP}$$

(8) $$R=\frac{TP}{TP+FN}$$

(9) $$F1=\frac{2*P*R\;}{\;P+R\;}$$

(10) $$mAP=\frac{\sum_{q=1}^{Q}AP(q)}{Q}$$

where precision signifies the ratio of accurately identified items in a project’s sample. Furthermore, recall denotes the connection between accurately identified entries and the total entries in the sample. *AP* stands for the average precision of distracted driving behavior in class *q*. Meanwhile, *mAP* calculates the mean average precision across all categories. Lastly, F1 represents the average of precision and recall. 

In addition to metrics related to accuracy, the lightweight evaluation of a model primarily focuses on the following three indicators: parameters, floating-point operations (FLOPs), and the size of model weight files. 

Parameters refer to the adjustable quantity of parameters within a model, which need to be fine-tuned during the training process to optimize model performance. Floating-point operations (FLOPs) are employed to gauge the computational complexity of a model. FLOPs represent the total number of floating-point calculations performed during the inference (forward propagation) stage, reflecting the computational resources required by the model. The size of the model weight files indicates the file space needed to store the trained model parameters. This holds significant importance for the deployment and transmission of the model.

FPS is introduced as an indicator of processing speed, where FPS stands for “frames per second”. This is a metric to measure the processing speed of algorithms, especially in real-time object detection tasks. A higher FPS value indicates a faster processing speed of the algorithm, allowing it to handle more image frames in a shorter amount of time.

### 3.3. Experimental Result Analysis

#### 3.3.1. Results before and after Training

To validate the performance of the proposed YOLO-LBS model, we conducted a comparative validation with the sub-network YOLOv8n on the same validation dataset after training. The specific results are presented in Table 1.

Table 1 illustrates that the YOLO-LBS model proposed in this study exhibits superior performance across various metrics including precision, recall, F1, and mAP, when compared with YOLOv8n (with increases of 2.8%, 1.7%, 2.3%, and 5.1% respectively). This advantage stems from the enhancements made to the BiFPN architecture, which augment the model’s feature-extraction capabilities for capturing distracted-driving behaviors across different scales. Additionally, the introduced attention mechanism effectively mitigates the interference caused by complex backgrounds in recognizing distracted driving behaviors. It is worth noting that the YOLO-LBS model proposed in this study reduced parameters by 39.3% and increased speed by 15.7%. This accomplishment can be attributed to the lightweight design of GhostConv, along with the innovative GhostC2f architecture, both of which significantly contribute to the reduction of model parameters. In summary, the model proposed in this study stands out in terms of both lightweight design and recognition accuracy when compared with the baseline model.

**Figure 11:**
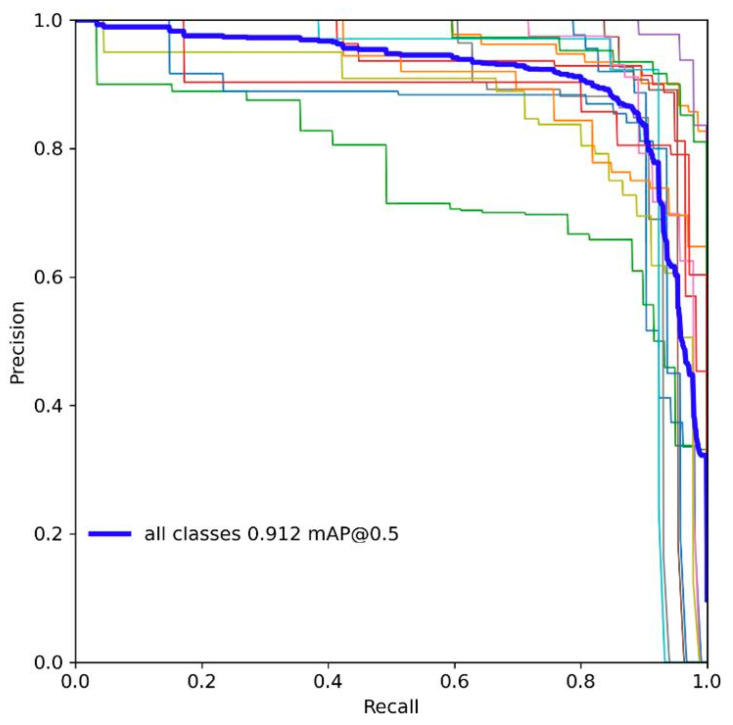
PR diagram of YOLOv8n.

**Figure 12:**
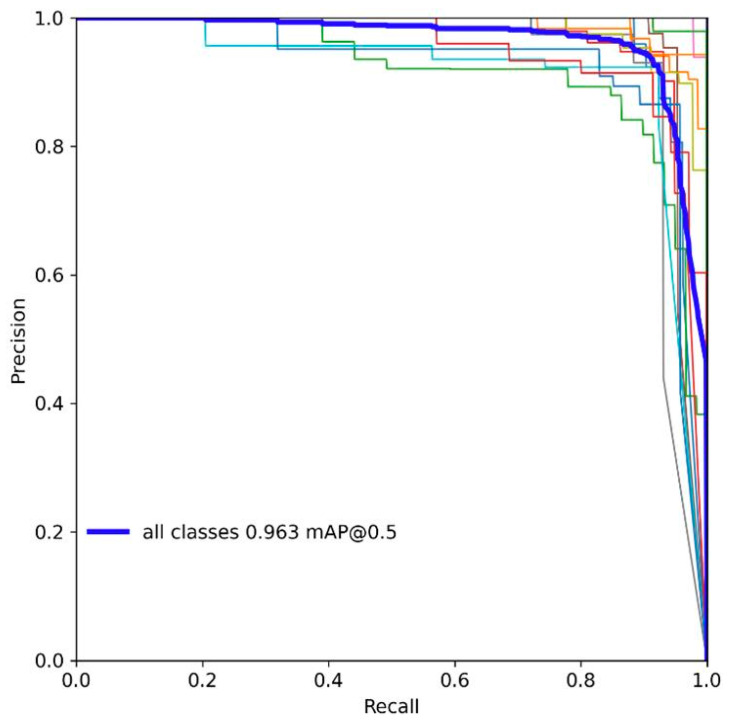
PR diagram of YOLO-LBS.

To assess the model’s performance with greater accuracy, during the testing phase, we plotted the PR curve of the model before and after improvement at an IOU of 0.5, as shown in Figure 11 and Figure 12. 

The area under the curve (AUC-PR) is commonly used as a metric to gauge model performance, where a larger AUC-PR indicates better performance across various precision–recall combinations. It is evident that the improved model has a higher AUC-PR.

#### 3.3.2. Ablation Experiments

**Figure 13:**
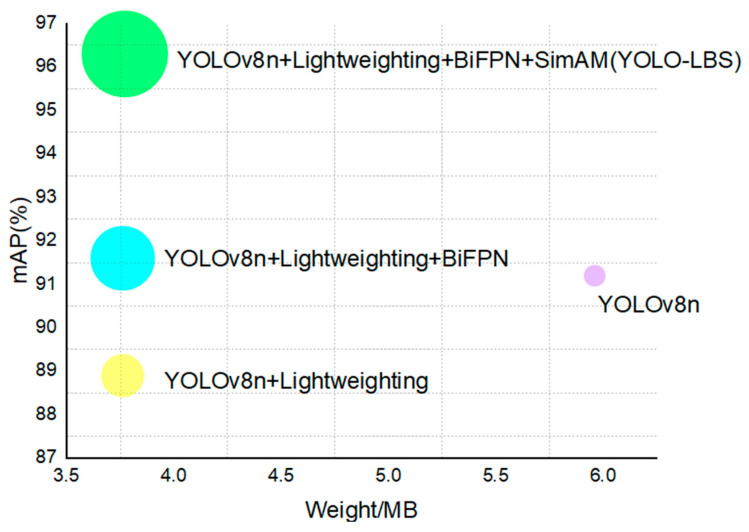
Comparison of mAP with different model weights.

To validate the effectiveness of the proposed enhancement approach, we conducted ablation experiments to gain a more intuitive understanding of the significance of each improvement method. The experimental results are comprehensively presented in Figure 13 and Table 2. 

Based on the data from Table 2 and Figure 13, a clear observation can be made that upon integrating the lightweight improvements with the sub-model YOLOv8n, the detection FLOPs and weight experienced reductions of 34.6% and 36.7%, respectively. The primary reason behind this phenomenon lies in the application of the GhostConv technique. This technique decomposes larger convolution kernels into multiple smaller ghost convolution kernels, thereby reducing the model’s parameter count and computational burden. Additionally, this study introduces the innovative GhostC2f design, which reduces the usage of conventional convolution operations and optimizes the weights among shared channels, further enhancing the model’s parameter configuration. Despite the lightweight operations having a certain impact on the model’s detection performance, they reduce the substantial weight and computational load of the model.

After the improvement of the BiFPN, the model’s mAP increased by 2.7%. This performance enhancement can be attributed to the introduction of the skip-connection mechanism in the BiFPN compared with the traditional FPN. This mechanism enables the simultaneous transmission of information in both top-down and bottom-up directions, thereby more effectively integrating features from different levels. This further enhances the model’s capability to fuse features related to distracted driving behaviors.

**Figure 14:**
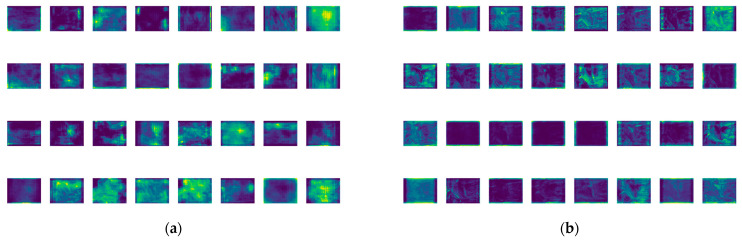
Feature visualization maps. (**a**) YOLOv8n + Lightweighting; (**b**) YOLOv8n + Lightweighting + BiFPN.

To conduct a more in-depth evaluation of the effects of the improved BiFPN, the models before and after the enhancement underwent feature-map visualization, as illustrated in Figure 14. From the visualization, it is evident that the feature maps generated by the BiFPN exhibit a more global and multi-scale representation of information. This aids the model in accurately capturing the feature representation of distracted driving behaviors across different scales.

Following the introduction of the SimAm mechanism, the model’s mAP value experienced an increase of 4.7%. This growth can be attributed to the feature of SimAm that assigns distinct weights to various input features, thereby further reinforcing the model’s capacity to extract relevant information effectively. 

**Figure 15:**
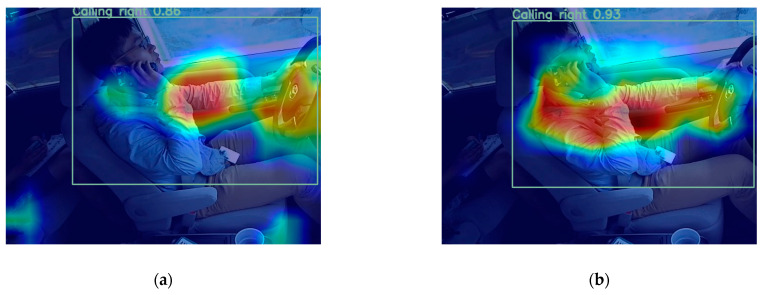
Grad-CAM visualization. (**a**) YOLOv8n + Lightweighting + BiFPN; (**b**) YOLO-LBS.

To thoroughly confirm the improvement achieved by the SimAm mechanism, we conducted Grad-CAM visualizations for the model before and after its integration. The results notably indicate that after the incorporation of the attention mechanism, the detection head becomes more focused on critical regions closely linked to distracted driving behavior. Consequently, the model’s capability to represent distracted driving behavior significantly improved, providing evidence of the effectiveness of the SimAm mechanism. Figure 15 is a feature-map visualization of the enhanced block, allowing clearer observation of the model’s enhanced feature-extraction impact.

#### 3.3.3. Mainstream Model Comparison Experiments

**Figure 16:**
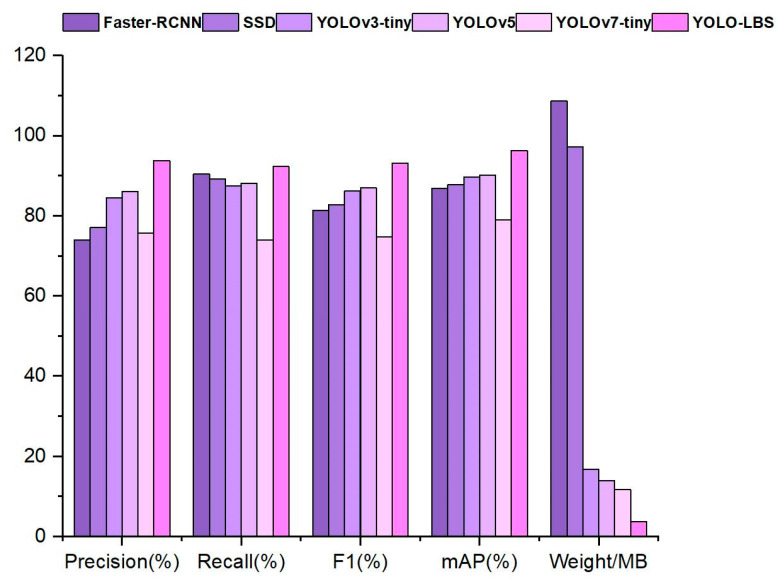
Comparison results with mainstream models.

To further validate the detection performance and lightweight advantages of the model proposed in this paper, a comparison was conducted with models such as Faster-RCNN, SSD, YOLOv3-tiny, YOLOv5, and YOLOv7-tiny. The detailed comparison results are shown in Figure 16 and Table 3. 

Analyzing the data in Table 3, it is evident that among numerous models, the Faster R-CNN model demonstrates comparatively lower detection accuracy. Moreover, this model necessitates the largest weight files, consequently demanding more computational resources and time for training and inference. In comparison to other single-stage object-detection models, Faster R-CNN fails to exhibit notable advantages. The SSD model also falls short in terms of detection accuracy and model weight. In contrast, YOLOv3-tiny, functioning as a lightweight model, boasts lighter weight. Its multi-scale prediction layers contribute to capturing objects of varying scales, thereby yielding superior detection performance. Noteworthy is the fact that YOLOv5, an evolution of YOLOv3-tiny, introduces data augmentation and the focus structure to attain more enriched channel information. As for YOLOv7-tiny, it possesses smaller model weight compared with YOLOv5, albeit sacrificing a certain degree of perceptual capability. The proposed YOLO-LBS model in this paper achieves lightweight architecture while simultaneously enhancing the model’s feature fusion and extraction capacities for objects of diverse scales. In summary, taking into account the balance between detection speed and accuracy, the algorithm proposed in this paper achieves the highest mAP value of 96.3%, while also having the fastest detection speed of 75.76 fps.

#### 3.3.4. Real-Vehicle Experiments

**Figure 17:**
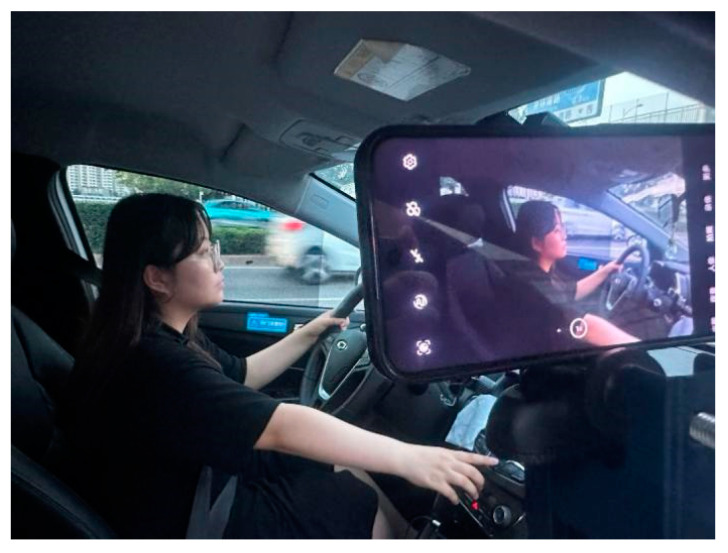
Data-collection procedure.

To assess the practical detection performance of the model proposed in this paper, a real-vehicle validation was conducted in May 2023 on Dagunan Road, Jinnan District, Tianjin. This experiment encompassed both daytime and nighttime periods, with a total of 10 drivers participating. During the experiment, the Guojin Junxing vehicle was employed as the experimental vehicle. Data pertaining to distracted driving behaviors from these 10 drivers were collected, and a test dataset containing 660 instances of distracted driving behaviors was constructed during 30 real-vehicle tests. The data-collection process is illustrated in Figure 17, and the test results are presented in Table 4.

**Figure 18:**
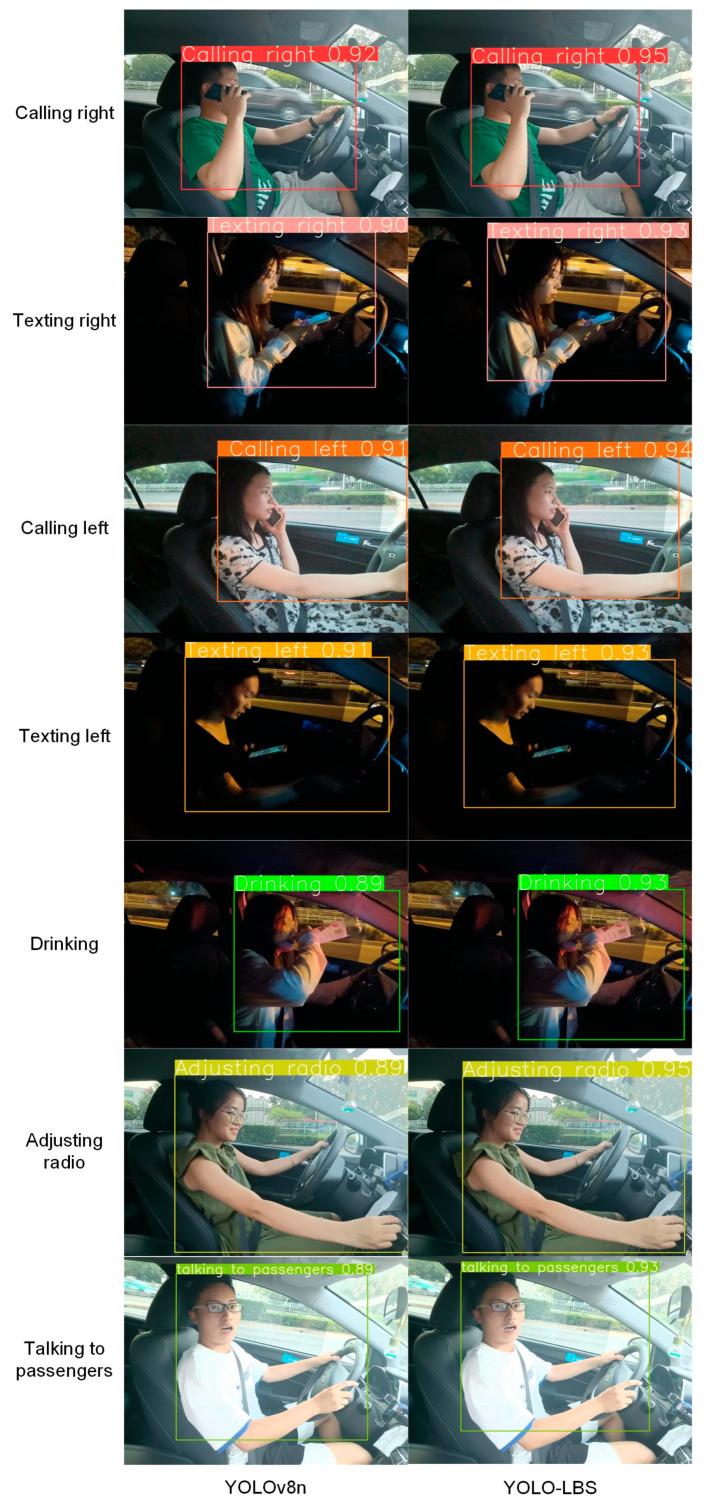
Test results of seven distracted driving behaviors.

**Figure 19:**
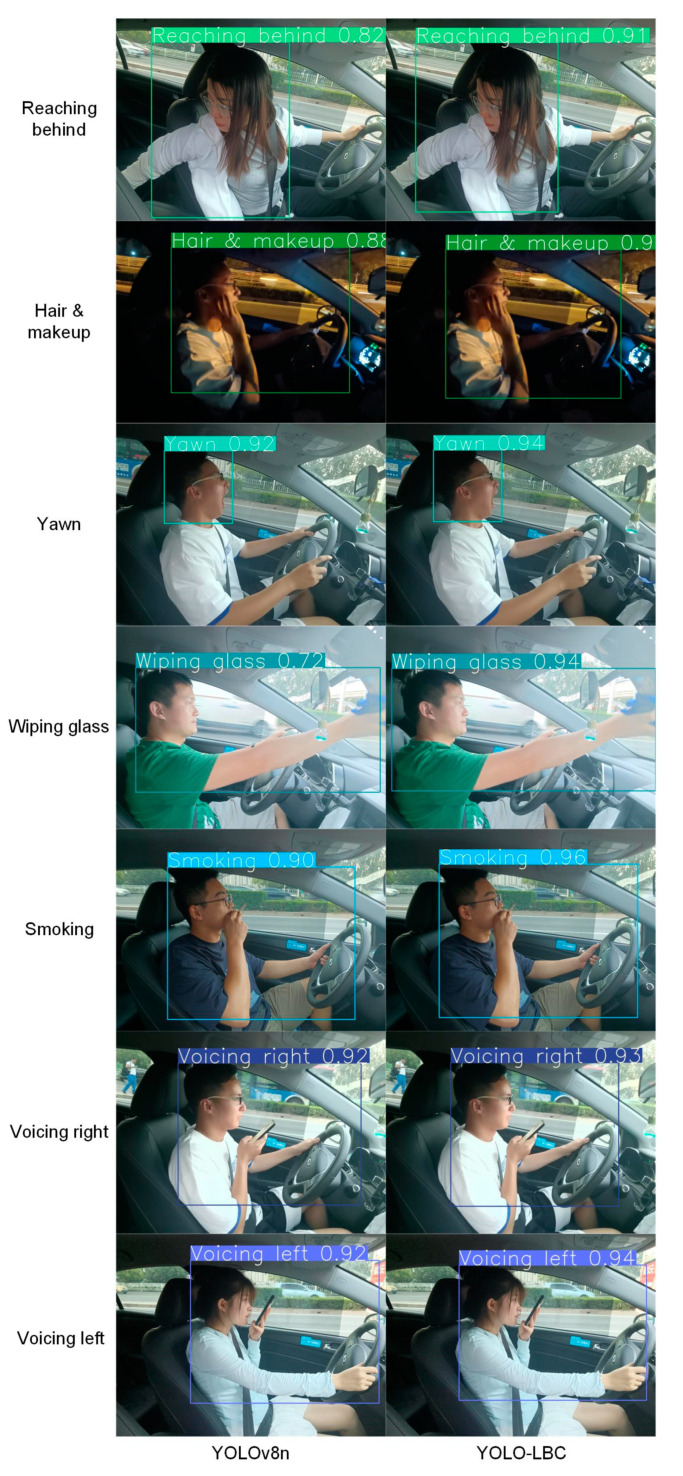
Test results of another seven distracted driving behaviors.

From Table 4, it is evident that the model proposed in this study achieved detection accuracies of 91.9% during the day and 90.3% at night. To visually showcase the adaptability of our model, selective instances of distracted driving behaviors were subjected to detection, and the results are depicted in Figure 18 and Figure 19. It is readily apparent that the model proposed in this study excels in identifying distracted driving behaviors, accurately discerning a variety of such behaviors with a high degree of confidence.

However, it is important to note that there exists variability in confidence levels among different distracted driving behaviors. For instance, actions such as using the right hand to hold a phone, adjusting the radio, and speaking with the left-hand exhibit distinct features, making them easier for the model to identify, resulting in higher confidence levels. Throughout the recognition process for these behaviors, the model demonstrates elevated accuracy and certainty.

## 4. Discussion

Timely detection of distracted driving behavior is crucial for reducing the occurrence of traffic accidents. Therefore, this paper proposes a model called YOLO-LBS, which outperforms the latest YOLOv8 model in distracted-driving-behavior detection, achieving more accurate detection results.

As the latest single-stage object detection model, YOLOv8 possesses excellent feature-extraction capability, resulting in remarkable object detection performance. However, its high performance comes with a substantial demand for computational resources, making it less suitable for deployment on in-vehicle terminals. Thus, this paper combines the GhostConv and YOLOv8n sub-models to improve computational efficiency while maintaining convolutional performance. Additionally, the GhostC2f structure is introduced to reduce model parameters and computation by eliminating traditional 1 × 1 and 3 × 3 convolutions. Furthermore, improvements have been made to the PAN structure to enhance multi-level feature fusion, thereby improving detection performance. Moreover, the novel SimAM has been introduced to further enhance the model’s information extraction capacity. Comprehensive experimental results in Table 1, Table 2 and Table 3 leads to the conclusion that the proposed model demonstrates superior performance in distracted-driving-behavior detection.

Compared with previous research methods, this paper employs computer-vision techniques which, in contrast to traditional on-site manual detection and physiological-signal sensing, more efficiently and rapidly detect distracted driving behavior. In the context of deep learning-based distracted-driving-behavior detection, numerous researchers have proposed various network structures [35,36,37], such as the two-stage Faster-RCNN algorithm. Although these algorithms have enhanced the performance of distracted-driving-behavior detection to some extent, their inference speed is slow, they have high parameter counts, and deployment costs are high. Furthermore, they have not effectively addressed issues related to different-scale behaviors and complex background interference. Although LightAnomalyNet proposed by Mehmood et al. [38] has made progress in improving detection speed, there is still room for improvement in accuracy. The YOLO-LBS model proposed in this paper not only balances detection speed and performance, but also enhances the capability for distracted-driving-behavior detection at different scales. Additionally, the proposed model more effectively recognizes distracted driving behavior in complex backgrounds, resolving the problem of increased parameters for efficiency improvement and introducing new perspectives to the field of distracted-driving-behavior detection.

Lightweight operations aim to reduce computational overhead, especially for real-time applications or devices with limited computational resources. However, by reducing the convolutional computations of the model, its ability to extract relevant information is diminished. In the future, we will consider integrating knowledge-distillation techniques, where knowledge from larger, more accurate models is used to train lightweight models. Additionally, we will incorporate the concept of attention mechanisms to improve detection accuracy. Our current dataset primarily consists of images taken under normal weather conditions. As a result, the model may not perform optimally under adverse weather conditions such as fog, rain, or snow. To address this limitation, we plan to augment our dataset with images captured under various adverse weather conditions. This can be achieved either by collecting new data or by employing synthetic data-augmentation techniques that simulate different weather conditions.

In the future, to ensure our model can be effectively deployed on vehicular devices with limited computational resources, our aim is to explore model compression techniques, such as quantization and pruning, to further reduce the size of the model’s parameters and weight files. Considering the latency-sensitive nature of vehicular applications, we are also contemplating the use of edge-computing solutions. This would bring data processing closer to the data source, ensuring faster response times. Additionally, to enhance the model’s detection performance, we are considering integrating the concept of attention mechanisms with lightweight network architectures, improving detection performance without increasing computational costs.

## 5. Conclusions

In this study, a novel computer-vision approach is proposed, named YOLO-LBS, for the rapid detection of distracted driving behavior. This approach combines two sub-networks, namely GhostConv and YOLOv8n, to synergistically leverage their respective advantages. GhostConv is employed for streamlining the network architecture, while YOLOv8n is utilized for extracting local intricate features. Furthermore, SimAM is integrated into the detection layer of the proposed model to enhance the significance of feature maps crucial for segmentation. Ultimately, the traditional SFDDD method has been expanded to encompass 14 distinct scenarios. Research findings demonstrate that the YOLO-LBS model achieves a reduction of 36.7% in weight, a 34.6% decrease in computational load, and a 5.1% enhancement in mAP. Additionally, a comparative analysis with mainstream object detection algorithms yielded satisfactory outcomes. However, lightweighting the model may lead to a decrease in model accuracy, and the dataset does not consider adverse weather conditions. Looking ahead, our future efforts will focus on further enhancing the detection speed and accuracy of the model, with plans to deploy it in vehicular terminal devices.

## Figures and Tables

**Table 1:** Comparison of distracted-driving-detection results.

Model	Precision (%)	Recall (%)	F1 (%)	mAP (%)	Parameters	FPS (f/s)
YOLOv8n	91.0	90.6	90.8	91.2	3008378	65.50
YOLO-LBS	93.8	92.3	93.1	96.3	1826646	75.76

**Table 2:** Results of the ablation experiments.

Model	mAP (%)	Recall (%)	FLOPs/G	Weight/MB	Parameters
YOLOv8n	91.2	90.6	8.1	5.96	3008378
YOLOv8n + Lightweighting	88.9	89.9	5.3	3.76	1821750
YOLOv8n + Lightweighting + BiFPN	91.6	90.4	5.3	3.76	1821758
YOLOv8n + Lightweighting + BiFPN + SimAM(YOLO-LBS)	96.3	92.3	5.3	3.77	1826646

**Table 3:** Comparison Results with Mainstream Models.

Model	Precision (%)	Recall (%)	F1 (%)	mAP (%)	Weight/MB	FPS (f/s)
Faster-RCNN	73.9	90.4	81.3	86.9	108.63	10.24
SSD	77.1	89.2	82.7	87.8	97.22	26.78
YOLOv3-tiny	84.4	87.5	86.2	89.6	16.69	33.43
YOLOv5	86.0	88.1	87.0	90.2	13.83	47.32
YOLOv7-tiny	75.6	74.0	74.8	79.0	11.79	58.54
YOLO-LBS	93.8	92.3	93.1	96.3	3.77	75.76

**Table 4:** Daytime and nighttime real-world test results.

Model	Precision (%)	Recall (%)	F1 (%)	mAP (%)
YOLO-LBS (daytime)	91.9	90.1	91.0	94.2
YOLO-LBS (nighttime)	90.3	89.6	89.9	93.4

## Data Availability

The data used in this study can be obtained from the corresponding authors.
